# Impact of Ileocecal Resection and Concomitant Antibiotics on the Microbiome of the Murine Jejunum and Colon

**DOI:** 10.1371/journal.pone.0073140

**Published:** 2013-08-27

**Authors:** Anthony A. Devine, Antonio Gonzalez, K. Elizabeth Speck, Rob Knight, Michael Helmrath, P. Kay Lund, M. Andrea Azcarate-Peril

**Affiliations:** 1 Department of Cell Biology and Physiology, School of Medicine, University of North Carolina at Chapel Hill, Chapel Hill, North Carolina, United States of America; 2 Department of Computer Science, University of Colorado at Boulder, Boulder, Colorado, United States of America; 3 Department of Surgery, School of Medicine, University of North Carolina at Chapel Hill, Chapel Hill, North Carolina, United States of America; 4 Department of Chemistry and Biochemistry, University of Colorado at Boulder, Boulder, Colorado, United States of America; 5 Howard Hughes Medical Institute, Boulder, Colorado, United States of America; 6 Microbiome Core Facility, School of Medicine, University of North Carolina at Chapel Hill, Chapel Hill, North Carolina, United States of America; Ludwig-Maximilians-University Munich, Germany

## Abstract

Ileocecal resection (ICR) is a commonly required surgical intervention in unmanageable Crohn’s disease and necrotizing enterocolitis. However, the impact of ICR, and the concomitant doses of antibiotic routinely given with ICR, on the intestinal commensal microbiota has not been determined. In this study, wild-type C57BL6 mice were subjected to ICR and concomitant single intraperitoneal antibiotic injection. Intestinal lumen contents were collected from jejunum and colon at 7, 14, and 28 days after resection and compared to non-ICR controls. Samples were analyzed by16S rRNA gene pyrosequencing and quantitative PCR. The intestinal microbiota was altered by 7 days after ICR and accompanying antibiotic treatment, with decreased diversity in the colon. Phylogenetic diversity (PD) decreased from 11.8 ± 1.8 in non-ICR controls to 5.9 ± 0.5 in 7-day post-ICR samples. There were also minor effects in the jejunum where PD values decreased from 8.3 ± 0.4 to 7.5 ± 1.4. PCoA analysis indicated that bacterial populations 28 days post-ICR differed significantly from non-ICR controls. Moreover, colon and jejunum bacterial populations were remarkably similar 28 days after resection, whereas the initial communities differed markedly. *Firmicutes* and *Bacteroidetes* were the predominant phyla in jejunum and colon before ICR; however, *Firmicutes* became the vastly predominant phylum in jejunum and colon 28 days after ICR. Although the microbiota returned towards a homeostatic state, with re-establishment of *Firmicutes* as the predominant phylum, we did not detect *Bacteroidetes* in the colon 28 days after ICR. In the jejunum *Bacteroidetes* was detected at a 0.01% abundance after this time period. The changes in jejunal and colonic microbiota induced by ICR and concomitant antibiotic injection may therefore be considered as potential regulators of post-surgical adaptive growth or function, and in a setting of active IBD, potential contributors to post-surgical pathophysiology of disease recurrence.

## Introduction

Crohn’s Disease (CD) and ulcerative colitis (UC) are two inflammatory bowel diseases (IBD), characterized by chronic inflammation of small bowel and/or colon (CD) [1,2]. Genetic susceptibilities, mucosal barrier defects [3,4], reduced ability to kill microorganisms with subsequent increased exposure of host T-cells to bacteria or bacteria products [5,6], host immune regulatory defects [1,7,8] and/or dysbiosis (altered microbiota) have roles in the pathophysiology of CD [9,10]. Approximately 80% of CD patients will require surgical bowel resection in their lifetime [11]. A common surgical intervention in CD involves the resection of the terminal ileum and cecum/proximal colon when medical therapies fail [12]. In CD and necrotizing enterocolitis (NEC), ileocecal resection (ICR) can be required to remove regions of seriously inflamed, fibrotic or necrotic bowel, and the need for recurrent or more extensive resections poses a risk of intestinal failure [13]. Complications that may be associated with ICR include the loss of ileum, which can reduce or prevent efficient reabsorption of bile acids, and the possibility that ICR may alter the microbiota in the jejunum or colon. Small intestinal bacterial overgrowth (SIBO) is common in CD, and more frequent in CD patients who had undergone surgery [14]. Patients with short bowel syndrome (SBS) due to multiple bowel resections frequently develop SIBO [15,16]. The overall qualitative and quantitative composition of the fecal microbiota of SBS patients compared with controls has been studied by temporal temperature gradient gel electrophoresis (TTGE) and qPCR [17]. The study showed that the microbiota of SBS patients was depleted in *Clostridium* *leptum* and *Bacteroidetes*, and enriched in *Lactobacillus* [17]. Given the frequency of ICR in CD or NEC, defining the impact of ICR on the resident microbiota is significant.

Non-pathogenic commensal gut microbiota have a profound impact on normal GI physiology. They ensure effective intestinal mucosal growth and immunity, and have an important role in nutrient digestion, absorption, angiogenesis, and fortification of the mucosal barrier. Additionally, bacteria promote host epithelial cell production of fucosylated glycans (on which many gut bacteria feed) [18]. Other functions of the GI microbiota include energy recovery from poorly digestible nutrients, modification of bile acids, and production of essential compounds not obtained in sufficient quantities through diet including folate and biotin [19,20].

The normal murine intestinal microbiota is dominated mainly by the phyla *Firmicutes* and *Bacteroidetes* [19,21,22], with a mucosa-associated bacterial population enriched in *Firmicutes*, predominantly of the families *Lachnospiraceae* and *Ruminococcaceae* [23]. In the present study, a mouse model of ICR previously developed by Dekaney et al. [24] was used to determine the impact of ICR on the microbiota in murine jejunum and colon. Other commonly used resection models include proximal small bowel resection in rat, pig or mouse models [25] but we developed the ICR model since ICR is a more frequent surgery in humans than proximal small bowel resection. An ICR model has also been developed in rats [26] but a mouse model has the potential advantage that it can be applied to genetically manipulated mice that develop spontaneous gastrointestinal diseases, such as IBD models [27]. The present study analyzed conventionally raised C57BL6 wild type mice after ICR to elucidate the impact of ICR and concomitant antibiotic dose on the microbiota in remnant jejunum and proximal colon in the absence of any ongoing disease. A combination of 16S rRNA gene pyrosequencing [28,29] and quantitative PCR (qPCR) was used to characterize the intestinal microbial communities over a time course before and after ICR. Mice given ICR were maintained on liquid diet for 4 days before and 7 days after ICR and were given a single antibiotic injection. Microbiota from non-operated controls given these same treatments were studied by qPCR to assess whether these treatments could contribute to observed changes in microbiota in jejunum or colon of animals given ICR. Our studies showed that ICR is followed by profound changes in both jejunal and colonic microbial communities, and some of the changes, particularly in colon although not jejunum were shared by animals given a single intraperitoneal injection of antibiotics.

## Materials and Methods

### Animal Housing and Treatment

**Figure 1:**
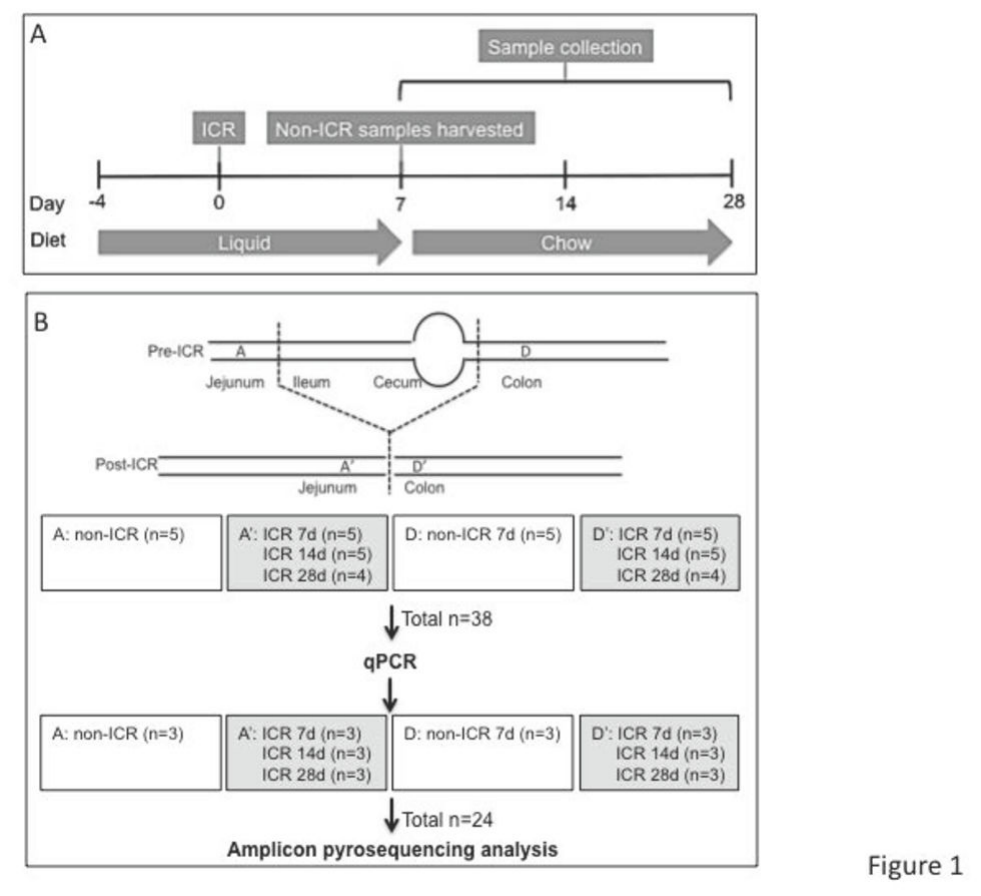
Scheme of sample collection intestinal sites before (top) and after resection (bottom). The number of mice (n) sampled in each location is indicated.

Adult 10-12 week male C57BL6 mice were purchased from Charles River Laboratories. Animals were allowed water ad libitum throughout experiments. Animals were transferred to a liquid diet (Micro-Stabilized Rodent Liquid Diet LAD 101/101A, Purina Mills) 4 days before the start of the study, and for 7 days after ICR (Figure 1). Liquid diet is usually given pre- and post-ICR because solid chow results in frequent obstruction and mortality while survival with the liquid diet protocol is >95%. Both non-ICR and ICR groups were given a liquid diet for this period to control for potential impact of liquid diet. Mice to be assigned to non-ICR and ICR groups were co-housed before surgery but after surgery ICR, mice were each housed separately in groups of 3 animals. Liquid diet consumption per individual animal was not measured. Liquid diet was replenished daily, and we observed no major difference in intake between non-ICR and ICR groups, although we cannot exclude reduced intake in the early period post-ICR. Immediately following surgery, a single injection of intra-peritoneal antibiotic Zosyn (Piperacillin and Taxobactam combination antibiotic) at a dose of 100mg/kg was administered to ICR mice. This broad-spectrum antibiotic was chosen since this is used clinically. Non-ICR controls did not receive antibiotics. A group of non-ICR and a group of ICR mice were killed at 7-day post ICR. Two other groups given ICR were returned to standard chow at day 8 following ICR and killed at 14 days and 28 days post ICR. ICR as performed as previously described [24]. Briefly, the small bowel was divided 12 cm proximal to the ileocecal junction and 1 cm distal to the cecum in the ascending colon. The mesentery of the resected intestine was ligated, resulting in the removal of 12 cm of the intervening ileum, cecum, and proximal right colon with reanastomosis to restore intestinal continuity. Initial non-ICR controls analyzed in parallel with ICR samples did not receive antibiotic. This was because we reasoned that a single injection of systemic antibiotic would be unlikely to affect luminal bacteria characterized 7-28 days later. However additional control experiments conducted specifically to assess the potential impact of a single antibiotic injection did reveal changes in total and specific bacteria and therefore the findings in ICR groups reflect the impact of both ICR and a single antibiotic dose. We note that patients given ICR would also receive systemic antibiotics. To assess the impact of a single antibiotic injection alone, mice received a liquid diet for 4 days and either intraperitoneal vehicle (non-ICR + vehicle) or the antibiotic (non-ICR + antibiotic), followed by 7 days of liquid diet exactly as for the 7-day post-ICR group. Additionally, since non-operated controls were initially sacrificed after the 11 days of liquid diet to match the liquid diet regime of the 7 day ICR group, we sampled luminal contents from additional non-operated controls given a liquid diet for 11 days followed by 7 or 21 days on normal chow to match the diet given to animals studied at 14 day and 28 days after ICR. These additional control samples and non-ICR, ICR+7, ICR+14 and ICR+28 groups were studied by qPCR for *Eubacteria, Firmicutes, Enterobacteriaceae*, and *Bacteroides* *-Porphyromonas-Prevotella* groups. The animal protocol was approved by the UNC Institutional Animal Care and Use Committee (IACUC).

### Sample Collection

Figure 1 summarizes the experimental design and sample collection during this study. Post-ICR samples of the luminal contents were collected from jejunum and colon at 7, 14 and 28 days following surgery. In mice that did not undergo ICR (non-ICR), luminal samples were collected from corresponding regions of jejunum and colon after 11 days on liquid diet. Approximately 200mg of luminal contents were collected at each site. The collected luminal contents from each sampling site were flash frozen in liquid nitrogen and stored at -80°C until processing.

### DNA Isolation

Isolation of total DNA was carried out on using a Qiagen BioRobot Universal (Qiagen) and the Qiagen Blood and Tissue Isolation kit. The Qiagen protocol was modified to ensure isolation of DNA from Gram positive as well as Gram negative bacteria as follows: approximately 100mg of lumen contents were resuspended in 100µl of 1X PBS with 8mg of lysozyme (Thermo, Fisher) and 200µl of ATL buffer in a 1.5ml microcentrifuge tube and incubated at 37°C for 30 minutes. Following the initial incubation, 20µl of proteinase K (Qiagen) were added and the mix was incubated overnight at 56°C. After the overnight digestion, tubes were centrifuged at 800g for 5 minutes. The supernatant was then transferred to a Qiagen S-block and sonicated in a VWR B2500A sonicator (VWR International) for 30 minutes at approximately 13 kHz at 65°C. Following the sonication step, the S-block was transferred to the bed of the BioRobot Universal and DNA isolation was carried out using a customized isolation protocol in the UNC Microbiome Core Facility. DNA was visualized by electrophoresis and quantified by PicoGreen (Invitrogen) using standard methods.

### Quantitative PCR

Quantitative PCR analysis was carried out on genomic DNA from samples isolated from lumen contents to verify changes in abundance of selected bacterial groups. Primers used in this study are listed in Table 1. Each qPCR reaction contained 10µl of 2X PowerSYBR Master Mix (ABI), 1µl of 10µM of each primer in the primer pair and 100ng of target DNA. The remaining volume was made up with PCR grade water. All qPCR reactions were carried out in triplicate in an ABI 7500 Fast thermocycler. Negative and positive controls were included in all amplification steps. Data were analyzed using the ABI 7500 Software version 2.0.1. The assay conditions were: initial hold at 50°C for 5 minutes, 95°C for an additional 10 minutes then 40 cycles of an initial denaturing step at 95°C for 1 minute, a primer binding step of 52°C for 2 minutes and a 1 minute extension at 72°C. Amplicon numbers were calculated during the annealing step, according to the software protocol. The correlation coefficients for standard curves were 0.99 and PCR efficiencies ranged from 93.7% to 99%. Melting curve analysis was performed to determine sample quality. The absolute quantification of DNA molecules was performed according to Pfaffl [30]. Standard curves for each primer were constructed using known quantities (molecules/ng DNA) of a standard DNA samples, which was a size-selected and purified PCR product of the amplification of the 16S gene from a random mouse sample. The linear equation for the standard curve (i.e., for preparations containing known quantities of DNA) was then used to interpolate the number of copies present in each unknown sample. One-Way ANOVA using index data mode was performed in Origin (OriginLab) to test whether or not the means of samples were equal or statistically different. The natural logarithm of the number of molecules of DNA encoding the16S rRNA gene was used for calculations, and Tukey tests were used for pairwise comparisons.

**Table 1:** qPCR primers used in this study.

Primer	Sequence (5' to 3')	Target	Reference
Uni 331F	TCCTACGGGAGGCAGCAGT	*Eubacteria*	[59]
Uni 797R	GGACTACCAGGGTATCTAATCCTGTT		[60]
FirmF	GGAGYATGTGGTTTAATTCGAAGCA	*Firmicutes*	[61]
FirmR	AGCTGACGACAACCATGCAC		
EnteroF	CATTGACGTTACCCGCAGAAGAAGC	*Enterobacteriaceae*	[62]
EnteroR	CTCTACGAGACTCAAGCTTGC		
Bac 708F	CACGAAGAACTCCGATTG	*Bacteroides* *–Porphyromonas–Prevotella*	[63]
Bac 1080R	CACTTAAGCCGACACCT		[64]

### 16S rRNA bacterial tag-encoded pyrosequencing

Initial amplification of the V1-V3 region of the bacterial 16S rDNA was performed on 24 individual samples from the study (n=3 per region or condition randomly selected from the full sample set). Master mixes for these reactions utilized the Qiagen Hotstart Hi-Fidelity Amplification Kit with a forward primer composed of the Roche Titanium Fusion Primer A (5’-CGTATCGCCTCCCTCGCGCCATCAG-3’), a 10 base pair MID bar code (Roche) that was unique to each of the samples processed, and the universal bacteria primer 27F (5’-AGAGTTTGATCCTGGCTCAG-3’) [28]. The reverse primer was composed of the Roche Titanium Fusion Primer B (5’-CTATGCGCCTTGCCAGCCCGCTCAG-3’) a 10 base pair MID identical to the forward primer and the reverse bacteria primer 338R (5’-TGCTGCCTCCCGTAGGAGT-3’) [28]. The thermal profile for the amplification of each sample was an initial denaturing step at 94°C for 5 minutes, followed by a cycling of denaturation at 94°C for 45 seconds, annealing at 50°C for 30 seconds, a 1 minute 30 second extension at 72°C (35 cycles), a 10 minute extension at 72°C and a final hold at 4°C. Each sample was gel-purified individually using the Qiagen Gel Extraction Kit (Qiagen), and the concentration was standardized. Equal amounts of the 16S rRNA gene amplicons from individual samples were bar-coded, pooled, and sequenced on a Roche GS FLX 454 sequencer (High Throughput Sequencing Facility, Chapel Hill NC) using the Titanium sequencing reagents and protocols. Sequence analysis was performed using Quantitative Insights Into Microbial Ecology (QIIME) [31] with default parameters, including removing sequence artifacts using Denoiser [32] and chimera removal with ChimeraSlayer; clustering via uclust [33] at 97% similarity; then classified taxonomically using the RDP classifier [34] retrained with Greengenes [35]. A single representative sequence for each OTU was aligned using PyNAST [36], then a phylogenetic tree was built using FastTree [37]. The phylogenetic tree was used for computing the UniFrac distances between samples [38].

### Amplicon data analysis

Sequencing data has been submitted to the National Center for Biotechnology Information (NCBI) under BioProject ID PRJNA208899. Downstream analysis of amplicon data was carried out using the QIIME pipeline. Sequences were first demultiplexed and assigned to their respective sample libraries based on the bar code identified within each sequence. Compiled phylogenetic information for each sample was used to determine the relative percentages of each bacterial phylotype in each sample. Phylogenetic trees were created in QIIME using FastTree, using PyNAST multiple sequence alignments. A random selection of 2,216 sequences from each sample was used for rarefaction analysis to ensure an even sampling depth. Non-parametric values; Phylogentic Diversity (PD), Richness and Chao 1 estimates for each sample were also calculated in QIIME using the default parameters within the alpha_diversity.py script. To evaluate the similarities between samples a combination of UniFrac significance, principal coordinate analysis (PCoA) using Fast UniFrac [39] and network analysis [40,41] was performed to compare samples based on sample site, location, and time after ICR.

## Results

### Quantitative PCR

**Figure 2:**
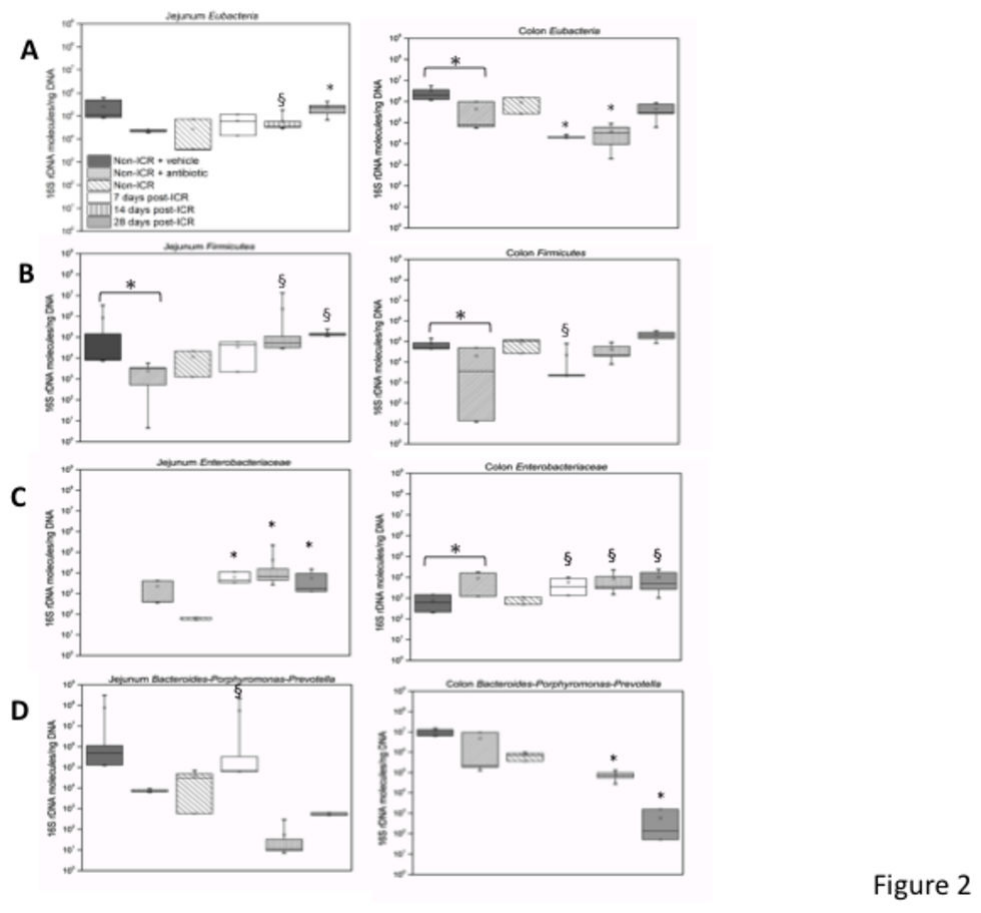
Quantitative PCR (qPCR) analysis of Eubacteria, Firmicutes, Enterobacteriaceae, and the Bacteroides-Porphyromonas-Prevotella group in jejunum and colon. Samples are: non-ICR+vehicle, animals received a liquid diet for 4 days before single injection vehicle and liquid diet for 7 days post-vehicle; non-ICR+antibiotic, animals received a liquid diet for 4 days before single antibiotic injection and liquid diet for 7 days post-antibiotic; non-ICR, animals were fed a liquid diet for 11 days but did not receive antibiotics; 7 days post-ICR, animals received a liquid diet for 4 days before ICR and concomitant single antibiotic dose, and liquid diet for 7 days post-ICR when they were sacrificed; 14 days post-ICR, animals received a liquid diet for 4 days before ICR and concomitant antibiotic dose, a liquid diet for 7 days post-ICR and then switched to a standard diet for 7 more days when they were sacrificed; 28 days post-ICR, animals received a liquid diet for 4 days before ICR and concomitant antibiotic dose, and liquid diet for 7 days post-ICR and then switched to a standard diet for 21 more days when they were sacrificed. Values along the y-axis are mean ± SEM numbers of molecules of targeted 16S rDNA per nanogram of DNA. ANOVA followed by Tukey’s tests for pairwise comparisons at each time point versus the non-op controls and between antibiotic control and antibiotic groups were performed. Symbols indicate * P ≤ 0.05 and § P ≤ 0.1.

Figure 1 outlines the experimental design and sampling sites in animals subjected to ICR, and in non-ICR controls. Post-ICR samples were collected at 7, 14 and 28 days following surgery as indicated. We selected day 7 after ICR because at this time point we have previously reported that the expansion of crypts that contribute to long term adaptation is initiated along with acute changes in proliferation and crypt depth and villus height [24,27,42]. At later time points of 14 and 28 days, the acute increases in proliferation, crypt depth and villus height are decreasing whereas sustained increases in the overall number of jenunal cypts persist [24]. Therefore, these time points allow for the comparison of sustained changes in the microbiota during both the acute and sustained adaptive response in our animal model. DNA samples were prepared from intestinal lumen contents of wild type C57BL6 mice including non-ICR controls and mice at 3 different time points after ICR. All DNA samples were subjected to qPCR analysis. Quantitative PCR revealed that at 7-28 days after ICR and a single intraperitoneal antibiotic injection given at surgery in both jejunum and colon there were significant changes in total bacterial populations as well as changes in specific bacterial groups including *Firmicutes*, *Enterobacteriaceae*, which is a family within the phylum *Proteobacteria*, and the *Bacteroides* *-Porphyromonas-Prevotella* group (Figure 2). The highest bacterial load was detected in non-ICR colon samples. In general, the total number of DNA molecules encoding 16S rRNA genes, which can be correlated with total bacterial load, and the abundance of specific DNAs encoding16S rRNA genes that correspond to specific bacterial groups, was higher in colon than jejunum at least by an order of magnitude. Seven days after ICR, the total bacterial load detected by Eubacterial primers in the colon decreased dramatically, by almost two orders of magnitude (Figure 2A); however, by 28 days post-ICR, the total bacterial load in the colon increased to values approaching the non-ICR values. The lowest bacterial load was detected in the jejunum of non-ICR controls. Overall, we observed a trend for increases in the total bacterial load in the jejunum following ICR, with abundance of jejunal *Eubacteria* 28 days after ICR approaching the levels found in the colon of non-ICR controls and in 28-day ICR colon samples.

With regards to specific taxa, in the colon the abundance of Firmicutes decreased at 7 days post-ICR compared to non-ICR controls, but approached non-ICR levels by 14 days and was similar to non-ICR levels by 28 days. In the jejunum, there was a progressive rise in the abundance of Firmicutes, so that levels were similar to those observed in the post-ICR colon by 28 days after ICR (Figure 2B). *Enterobacteriaceae* showed a different pattern, with initial levels lower in non-ICR samples from both jejunum and colon compared to any of the post-ICR timepoints (Figure 2C). Quantitative PCR showed that levels of *Enterobacteriaceae* in both jejunum and colon significantly increased by 7 days after ICR and were then maintained at 14 and 28 days. Abundance of the *Bacteroides* *-Porphyromonas-Prevotella* group decreased significantly in colon by 14 days after ICR and showed an even more dramatic decrease 28 days after ICR. Despite several attempts, we failed to detect this bacterial group in 7-day post ICR samples from the colon (Figure 2D). The jejunum showed a transient increase in the abundance of the *Bacteroides* *-Porphyromonas-Prevotella* group 7 days after ICR, with a significant decrease by 14 days, and an increase towards non-ICR values by 28 days after ICR.

Since the ICR group received an intraperitoneal (IP) injection of antibiotics before resection we also assessed the impact of either a single antibiotic or a vehicle injection on *Eubacteria, Firmicutes, Enterobacteriaceae* and the *Bacteroides* *-Porphyromonas-Prevotella* groups in the jejunum and colon of non-ICR controls at 7 days after antibiotic or vehicle. This revealed that in the colon, antibiotic administration resulted in significant decreases in *Eubacteria, Firmicutes*, a non-significant trend for decreases in *Bacteroides* *-Porphyromonas-Prevotella*, and a significant increase in *Enterobacteriaceae* (Figure 2). In jejunum, there were significant decreases in *Firmicutes* as a result of antibiotic and non-significant trends for decreased *Eubacteria* and *Bacteroides* *-Porphyromonas-Prevotella*. The *Enterobacteriaceae* in jejunum of mice that received the vehicle (non-ICR vehicle) were below the qPCR detection level. In colon of non-ICR mice given antibiotic, we observed similar directional changes in *Eubacteria, Firmicutes, Enterobacteriaceae and Bacteroides-Porphyromonas-Prevotella as* observed in colon of animals at 7 days after ICR plus antibiotic. In jejunum, antibiotic given to non-ICR animals did not elicit the same changes as observed in jejunum of animals at 7 days post-ICR and antibiotic. Thus we can conclude that a single antibiotic injection likely contributed to the changes in colon microbiota at 7 days post-ICR but cannot account for the impact of ICR on jejunal microbiota at 7 days post-ICR. Additionally, we analyzed whether the liquid diet for 11 days followed by return to chow as for 14 and 28 days post-ICR groups elicited changes in eubacteria or the specific bacterial groups. Liquid diet had no statistically significant impact on the studied bacterial taxa (data not shown).

### Amplicon analysis of the microbiota in non-ICR and ICR groups

**Figure 3:**
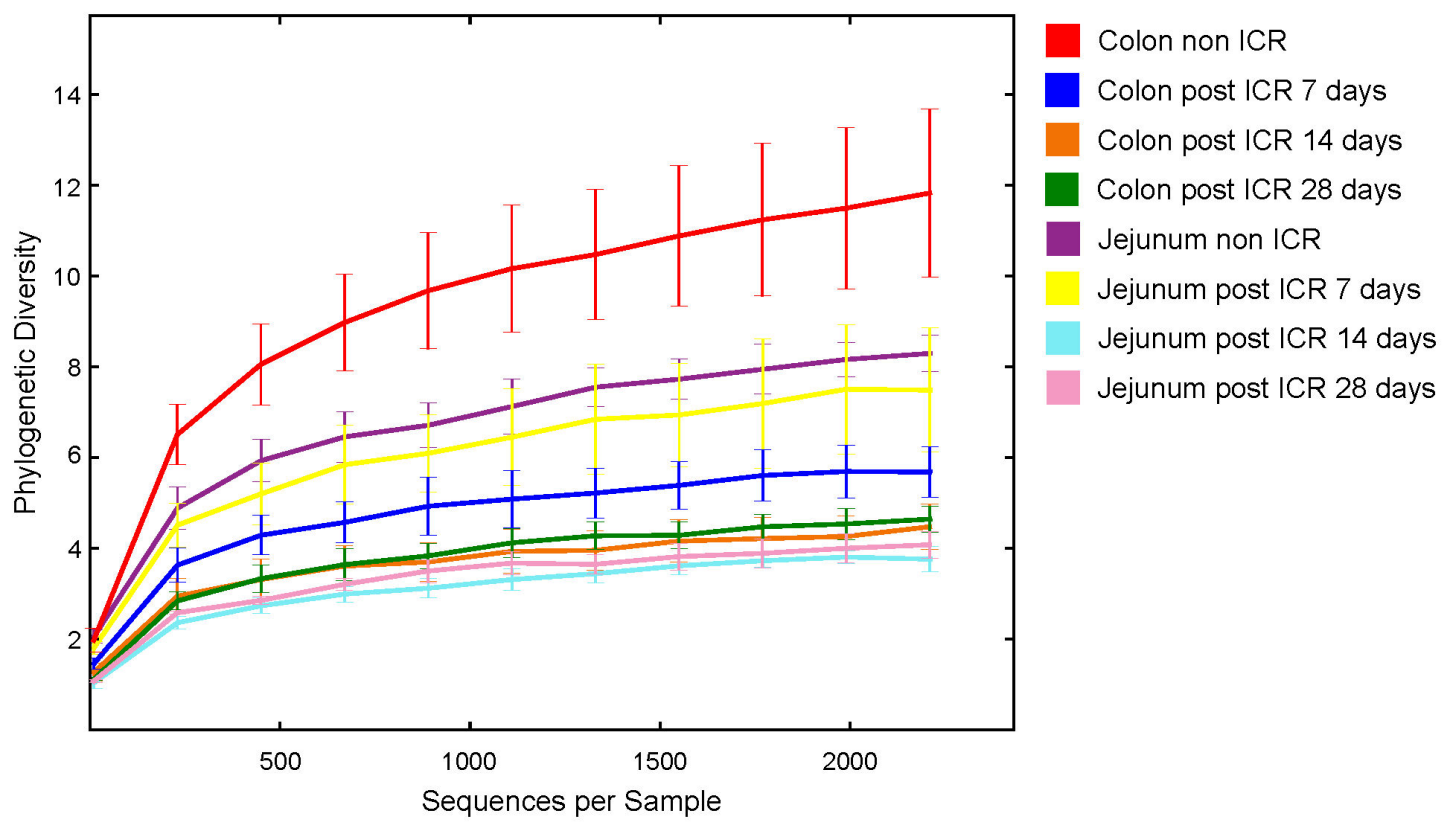
Rarefaction analysis of 16S libraries from non-ICR and post-ICR samples by location and time point. 2,210 16S rRNA gene sequences were randomly selected from each sample. Species were based on an Operational Taxonomic Unit (OTU) definition of 97% sequence identity.

**Figure 4:**
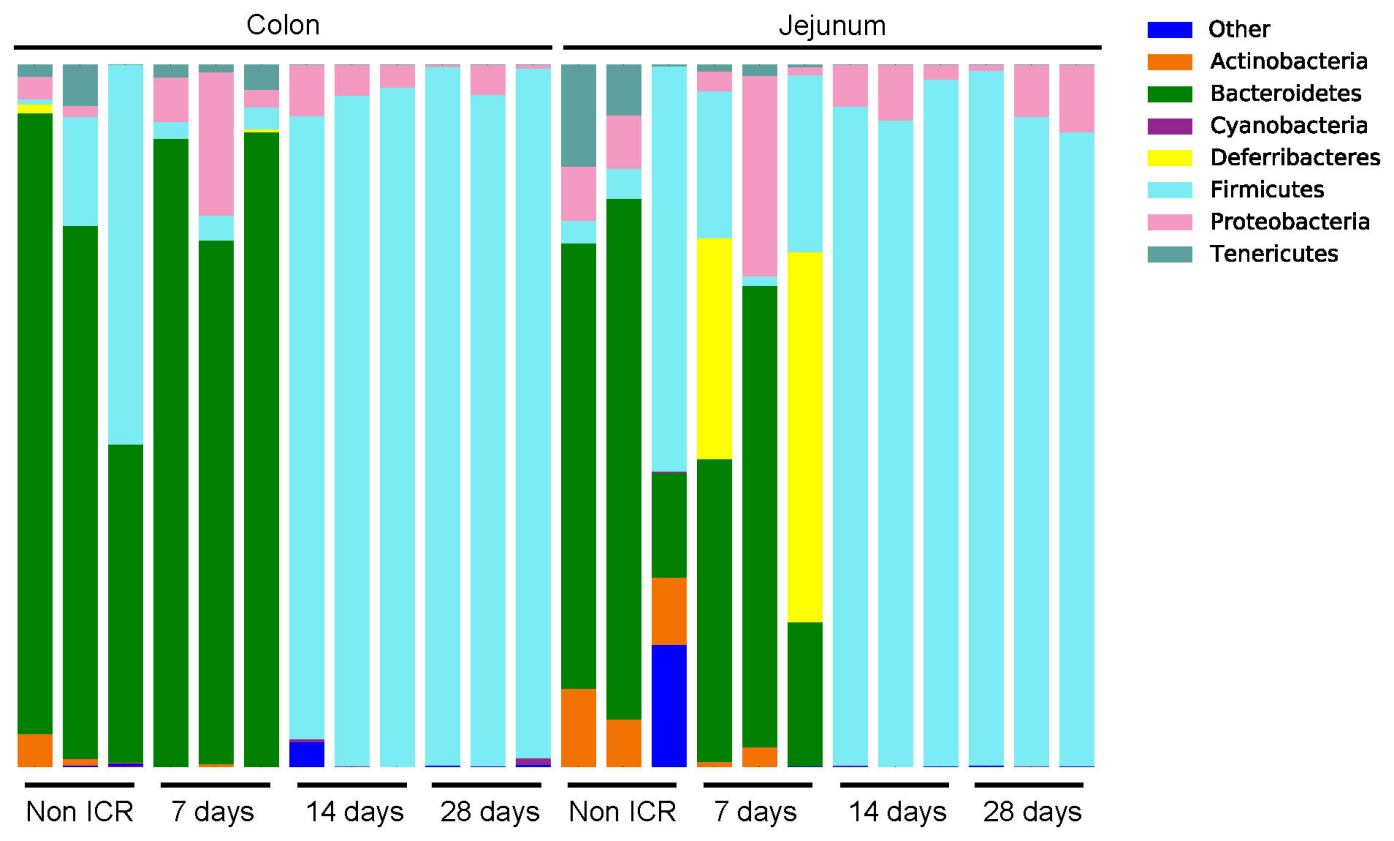
Relative abundance of bacterial phyla in non-ICR and post-ICR samples taken at 7, 14, and 28 days from jejunum and colon of rarefied samples at 2,210.

**Figure 5:**
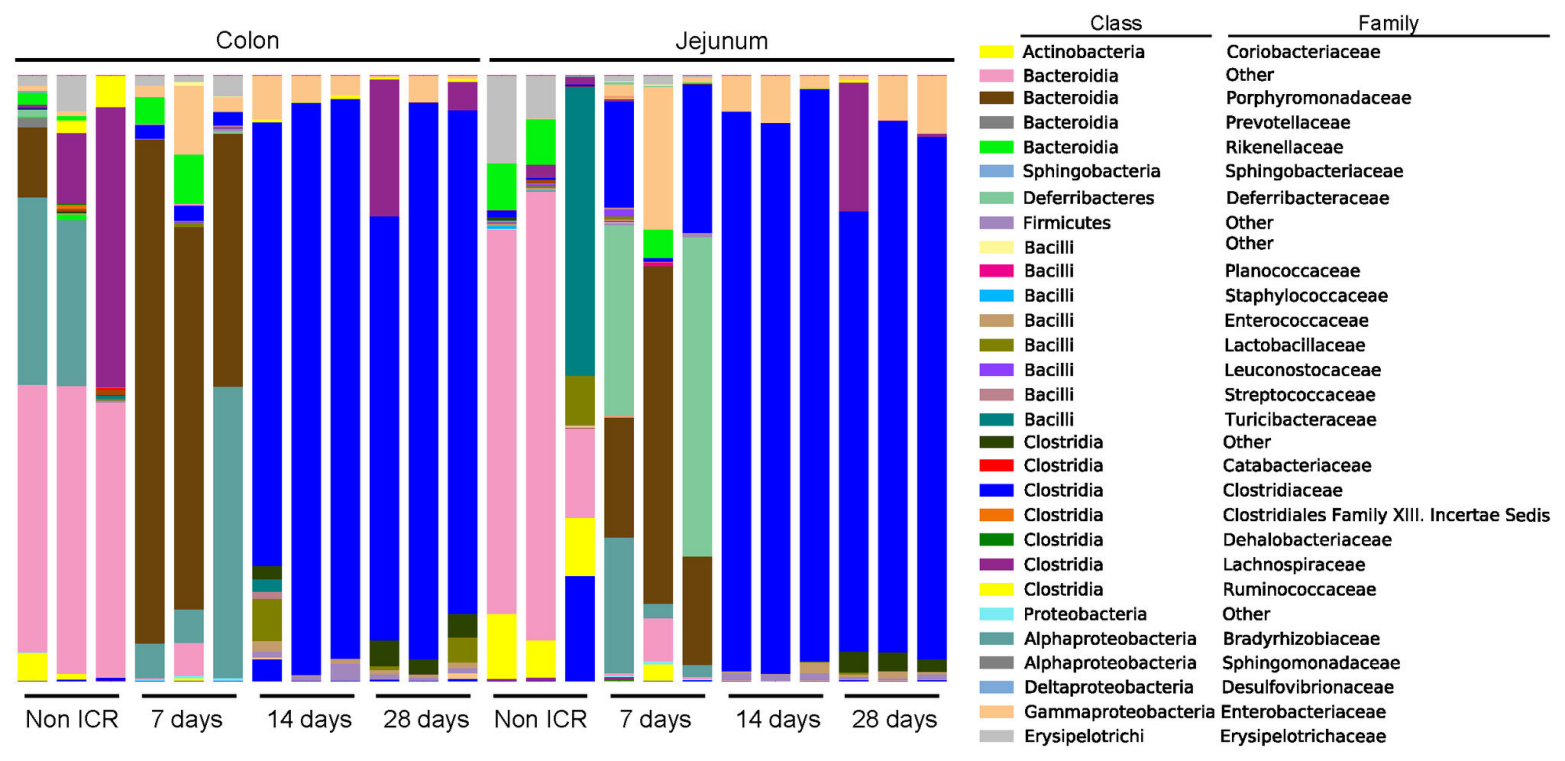
Relative abundance of bacterial families in non-ICR and post-ICR samples taken at 7, 14, and 28 days from jejunum and colon of rarefied samples at 2,210.

We selected 24 samples across jejunum and colon of non-ICR, ICR+7d, ICR+14d and ICR+28d for amplicon pyrosequencing, which permitted an analysis of the relative abundance of different bacterial taxonomic groups in 3 replicates per region and treatment group. A total of 311,951 sequences were assigned to 1,647 Operational Taxonomic Units (OTUs) at ≥ 97% similarity, clustering into 49 genera, 18 classes, and 9 phyla. Rarefaction analysis and diversity estimates were carried out to compare overall diversity of gut microbiota in non-ICR controls and ICR mice (Figure 3). Microbial population statistics of intestinal locations in ICR and non-ICR samples are shown in Table 2. Figures 4 and 5 show the relative abundance of bacterial phyla and families from the jejunum and colon of 3 different animals in each of the non-ICR and 7, 14, and 28 days post-ICR groups.

**Table 2:** Microbial population statistics of intestinal locations in non-ICR and post-ICR samples.

Location	Status	Time point (day)	Phylogenetic Diversity	Richness*	Average number of detected families
Jejunum	Non-ICR	7	8.30 (± 0.39)	73.10 (± 4.06)	16.00 (± 1.15)
	Post-ICR	7	7.49 (± 1.37)	57.96 (± 8.53)	20.00 (± 4.50)
	Post-ICR	14	3.76 (± 0.27)	49.80 (± 3.16)	8.00 (± 1.52)
	Post-ICR	28	4.08 (± 0.29)	55.53 (± 1.93)	8.33 (± 0,333)
Colon	Non-ICR	7	11.83 (± 1.85)	145.36 (± 35.42)	16.33 (± 2.02)
	Post-ICR	7	5.68 (± 0.55)	39.56 (± 3.67)	15.33 (± 2.18)
	Post-ICR	14	4.48 (± 0.50)	53.50 (± 2.22)	9.66 (± 1.20)
	Post-ICR	28	4.64 (± 0.28)	55.43 (± 2.77)	9.66 (± 0.66)

Three samples (n=3) were analyzed per time point and location.

^*^Average number of detected OTUs at a cutoff value of 97%.

### Amplicon analyses in jejunum and colon of non-ICR controls

In jejunum, non-ICR samples showed a predominance of the phyla *Firmicutes* (38% on average) and *Bacteroidetes* (35%). Interestingly, unclassified bacteria comprised 10% on average, but this was due to overrepresentation in a single sample. The phyla *Actinobacteria* and *Proteobacteria* were also represented (at 10 and 7% on average, respectively) (Figure 4). At the class level, *Bacteroidetes* (phylum *Bacteroidetes*) was the dominant class in two of the samples while *Erysipelotrichi* (phylum *Firmicutes*) was dominant in the third sample.

The colon of non-ICR controls contained a highly diverse microbiota (Table 2). Sequences from non-ICR colon samples showed that the predominant phylum detected in all three samples was *Bacteroidetes* (69.9% in average), followed by *Firmicutes* (23.4%) (Figure 4). Unclassified bacteria (amplicons classified as “Other”) comprised 0.3% of the total number of sequences for these samples. *Actinobacteria* and *Proteobacteria* were each detected at about 2% abundance. At the class level, we observed a dominance of *Bacteroidetes*, *Clostridia*, and unclassified *Bacteroidetes* (Figure 5).

### Impact of ICR and a single intraperitoneal antibiotic injection at surgery on microbiota of jejunum and colon

**Figure 6:**
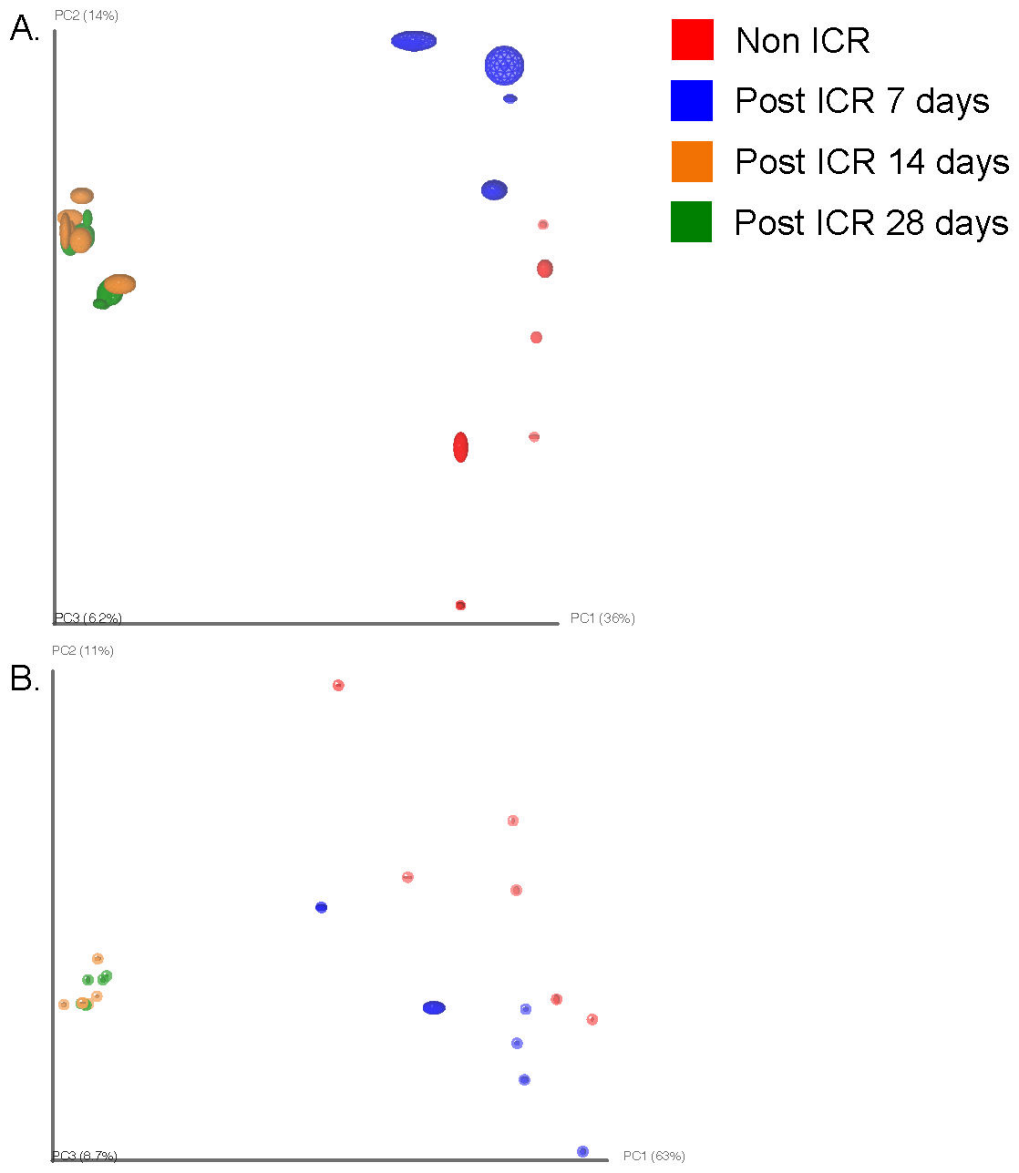
Jackknifing PCoA plots of unweighted (a) and weighted (b) UniFrac distance matrices of bacterial communities of intestinal samples from non-ICR and ICR from all time points. Point locations are the average location of 10 jackknife replicates using 2,210 random sequences per sample. Ellipses show the confidence based on these randomizations.

**Figure 7:**
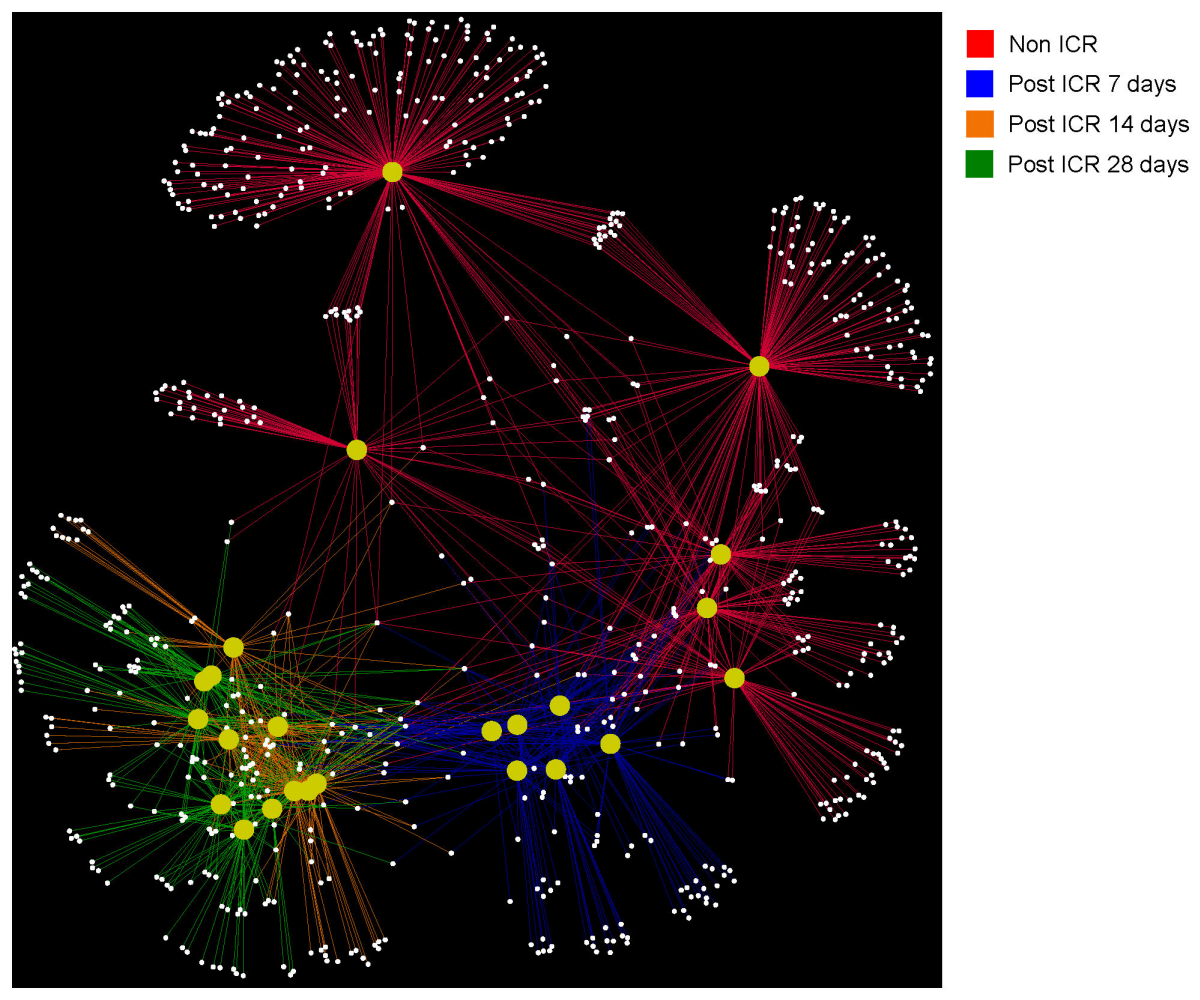
Network analysis of amplicon sequencing data revealing shared Operational Taxonomic Units (OTUs) between samples at different time points. Each yellow circle represents one sample. Lines are colored by time point (red are non-ICR samples, blue are 7-day post ICR samples, orange ar 14-days post ICR, and green are 28-days post ICR samples), and they represent OTUs shared by samples at different time points. Clustering shows that samples grouped by time point with samples from days 14 and 28 after ICR located in the same group.

Comparisons of data from jejunum and colon of non-ICR controls and animals at 7,14 or 28 days after ICR and a single antibiotic injection at surgery revealed significant changes in microbiota. Since colon of non-ICR animals given antibiotic alone showed significant and directionally similar changes in *Eubacteria* and *Firmicutes* as ICR animals given antibiotic, it is likely that changes in colon microbiota reflect at least in part, impact of antibiotics, even though antibiotics were given as a single systemic dose. Principal Coordinate Analysis (PCoA) of weighted and unweighted UniFrac [39] analysis of colon and jejunum amplicon sequences showed that samples clustered by sample day. Non-ICR and 7-day post-ICR samples clustered together, while samples taken at 14 and 28 days after resection were located within a tight group (Figure 6a and 6b). Additionally, we carried out a bipartite network analysis to further explore the interrelationship between sampling day and location, and shared and unique bacterial taxa. Nodes within this bipartite network were assigned to sample location and identified OTUs (≥ 97% similarity) within the samples. Edge coloration linking the nodes corresponds to the sampling time point. The network showed a clear separation of bacterial communities by sampling day (Figure 7). Non-ICR controls clustered together, and relatively close to samples taken 7 days after resection. However, samples from the 14 and 28 days time points were distinctively apart from the earlier time points and clustered together.

PD (phylogenetic diversity) decreased significantly (p < 0.05) in the colon, and decreased marginally in the jejunum, at 7 days after resection (Table 2). However, PD values increased in the jejunum and colon over time. Species richness in 7-day post-ICR samples from colon was notably lower than non-ICR or the other post-ICR samples (Table 2). Rarefaction curves were used to compare overall PD (i.e., the total tree branch length shared between OTUs) of each microbiome (Figure 3). PD in the jejunum and the colon after ICR was markedly lower than their respective non-ICR controls with rarefaction curves indicating that the total richness of the microbial community of non-ICR colon samples had not been completely sampled at the depth of coverage utilized.

### Impact of ICR on jejunal microbial communities

Jejunal microbial communities varied across the 3 animals 7 days post-ICR. Abundance of the phylum *Deferribacteres* (mainly members of the family *Deferribacteraceae*) increased dramatically in two samples (up to 52.7% in one of the samples), while *Proteobacteria* (mostly *Enterobacteriaceae*) increased in the other sample to 28.6%. A reduction in *Actinobacteria* (from an average of 9.1% in non-ICR samples to an average of 1.7%) was also observed at this time (Figures 4 and 5). Of interest was the increased abundance of *Porphyromonadaceae* within the phylum *Bacteroidetes* in jejunum at 7 days after ICR. In contrast, this phylum was mainly represented by the order *Bacteroidales* (families “other” and *Bacteroidaceae*), in non-ICR samples. The 14 and 28-day post ICR jejunum samples looked virtually identical and were composed almost exclusively of *Firmicutes* of the family *Clostridiaceae* and *Proteobacteria* of the family *Enterobacteriaceae*. However, *Actinobacteria* and *Bacteroidetes*, completely absent in 14-day samples, were detected at >1% abundance in samples taken 28 days after ICR (Figures 4 and 5).

### Impact of ICR on colon microbial communities

The pyrosequencing data showed that the colon was the most impacted at 7 days after ICR and a single intraperitoneal antibiotic injection (Table 2). The microbiota of colon samples at this time point showed a dramatic change in the composition of the phylum *Bacteroidetes* (increased to 84.7% abundance in average, from an average of 69.9% in non-ICR samples). *Bacteroidetes* in non-ICR samples were represented by the order *Bacteroidales* (families “other” and *Bacteroidaceae*), while in 7 days post-ICR samples *Bacteroidetes* were still predominant but primarily represented by the *Porhyromonadaceae* family. An increase in the abundance of *Proteobacteria* was also observed in the colon at 7 days post-ICR (9.78% abundance in average from 1.58% in non-ICR samples), as well as a reduction of *Firmicutes* and unclassified bacteria (Figures 4 and 5). In the non-ICR colon, the predominant class of *Firmicutes* identified was *Clostridia* (family *Lachnospiraceae*). However, 7 days after resection, abundance of *Bacilli* (various *Lactobacillales*), *Peptostreptococcaceae* and *Erysipelotrichi* had increased.

At 14 and 28 days after ICR, there was an almost complete depletion of *Bacteroidetes* (< 0.01% abundance) in the colon. There was also a dramatic increase in the number of *Firmicutes* (>90%). The predominant class within *Firmicutes* was *Clostridia*, but there were increases in the class *Bacilli* ( *Enterococcus* and *Lactobacillus* ) and family *Peptostreptococcaceae*, with a notable decrease in the numbers of *Erysipelotrichi*. Shifts also occurred within *Proteobacteria*, which declined in relative abundance over time in the colon after ICR (9.8% at 7 days, 5% at 14 days and 1.7% at 28 days post-ICR colon samples), with decreased beta-Proteobacteria and a continued predominance of *Enterobacteriaceae* (Figure 5).

## Discussion

Using a combination of culture-independent methods, this study demonstrated that ICR coupled with a single intraperitoneal injection of antibiotics had a major impact on the intestinal microbiota. There were changes in the microbiota of jejunum and colon following ICR, with a decrease in diversity, especially in the colon. PCoA plots of microbial communities from samples analyzed by pyrosequencing indicated that populations at 28 days after ICR differed considerably from non-ICR controls demonstrating long-lasting effects of ICR on microbial communities. Moreover, the bacterial populations of the jejunum and colon were nearly identical by the 14-day post-ICR timepoint. This was also observed by qPCR, where the total bacterial load in the jejunum increased over time after ICR, and reached higher bacterial numbers than non-ICR controls. In contrast, levels of *Eubacteria* in the colon decreased sharply immediately after ICR, and returned to near non-ICR levels by 28 days after ICR. These results are consistent with ICR in this murine model leading to small intestinal bacterial overgrowth (SIBO), a condition common in human patients who undergo ICR to treat CD [14]. In SIBO, loss of the cecal valve allows for colonic bacteria to infiltrate and colonize the small intestine [43]. Infiltration of colonic bacteria into the jejunum both increases the bacterial load in the jejunum and as observed by our pyrosequencing data, leads to the homogenization of the microbiota. However, our findings in the murine model indicate that early after ICR, in the first 7 days, there is a divergence of jejunal and colonic bacteria such that certain phyla like *Deferribacteres* were represented at high proportions in 2 of 3 jejunal samples, but were not detected in the colon. By 14 days and through 28 days after ICR, a relatively stable and high representation of the phylum *Firmicutes* was observed in both the jejunum and the colon, with *Proteobacteria* representing the other major phylum. It must be noted that at 7 days after ICR, mice were transferred from a liquid diet to a standard chow diet, which could contribute to compositional changes in the microbiota observed at 14 and 28 days after ICR. However, our qPCR data showed no significant differences in major bacterial taxa between animals fed the standard diet versus animals fed a liquid diet for 7 days and then the standard diet for 7 or 14 days (data not shown).

Although the intestinal microbiota returned towards a homeostatic state, with re-establishment of *Firmicutes* (*Clostridia*) as one of the predominant phyla in both the jejunum and the colon at 28 days after ICR and with *Proteobacteria* at low abundance, we did not detect *Bacteroidetes* in the colon at the 28-day post-ICR. At this 28-day timepoint, *Bacteroidetes* was only detected with an abundance of >0.01% in the jejunum. Therefore, ICR clearly had long-term effects on the proportion of the two major phyla (*Bacteroidetes* and *Firmicutes*) with a transient expansion of two minor phyla (*Proteobacteria* and *Deferribacteres* ) either present at a higher proportion due to depletion of the other microbes or overgrown due to the advantage of an altered, less complex niche. Persistent changes to the microbiota due to other perturbations have been reported in previous studies. Dethlefsen et al. [44] showed that repeated doses of the antibiotic ciprofloxacin resulted in a rapid decrease of diversity and shifts in the community composition of human fecal samples, but that subjects began to return to their initial state after 1 week. The return however was most often incomplete. Additionally, the microbiota of each participant in the study recovered at a unique rate. Antonopolous et al. [21] and Manichanh et al. [45] showed that between two weeks and one month after an antibiotic treatment in mice and rats respectively, the microbiota returned to a steady-state condition. This involved re-establishment of *Bacteroidetes* and *Firmicutes* as the predominant phyla, following an initial increase in *Proteobacteria*, and a return to similar total bacterial loads in the fecal samples [21,45].

QPCR data showed increased abundance of *Proteobacteria*, specifically of the family *Enterobacteriaceae* in jejunum and colon after ICR. The increase of the populations of *Proteobacteria* might be related to several factors. Immediately following surgery, before the abdominal cavity was sutured, a single dose of intra-peritoneal antibiotic was routinely administered to reduce the risks of acquired infections, affecting the viability of other members of the intestinal microbiota. This effect of the single antibiotic dose alone was demonstrated in our study, which showed increases in *Proteobacteria* in the colon of non-operated mice after antibiotic administration and decreased *Eubacteria* and *Firmicutes*. The other potential factor that might promote the early predominance of *Proteobacteria* could be due to the host immune response to surgery, with recruitment and infiltration of host-immune cells to the surgical site [46] and production of reactive oxygen and nitrogen species [47], which could have a more pronounced effect on the obligate anaerobic members of the microbiota. Similar results were previously reported in studies where the mammalian intestinal microbiota, when perturbed by antibiotic treatment, showed an overall decrease in total diversity [21,45,48,49] and increased numbers of *Proteobacteria* [21,45]. In the study by Antonopoulos et al. [21], mice given amoxicillin/metronidazole/bismuth for 10 days showed a massive increase in the abundance of *Enterobacteriaceae*, a gamma-Proteobacteria (75.5% of the sequences identified), in the cecum.

Implications of the expansion of the family *Porphyromonadaceae* within the phylum *Bacteroidetes* in both the jejunum and the colon 7 days after resection require further investigation. It could be speculated that members of this family are more resistant to the dramatic changes in the intestinal environment during or immediately after ICR, which potentially include increased oxygen due to the surgical intervention itself, increased bile acids reaching the colon (and indirectly the jejunum) due to loss of ileum or altered microbial populations themselves. Reduced populations of *Porphyromonadaceae* have been reported in mice in response to metronidazole treatment after *Citrobacter rodentium*-induced colitis [50], in response to a diet high in saturated fatty acids [51], and stressor exposure [52], which contrast with the early ICR-associated increases in this family found here.

ICR is commonly associated with complications including bacterial overgrowth, bile salt malabsorption, or short bowel syndrome, necessitating long-term intravenous nutrition [13]. Bile salt malabsorption is caused by the loss of the distal ileum and the ascending colon, which absorb bile salts under normal physiological conditions [53–55]. This malabsorption leads to a luminal environment rich in bile salts, which can affect survival of bile intolerant commensal microbiota, contribute to nutrient malabsorption, diarrhea and mucosal damage. Dekaney et al. [24] used the ICR mouse model to show that the remnant jejunum exhibits adaptive growth after ICR, resulting in an increase in crypt depth and villus height by 7 days and increase in mucosal circumference by 6 weeks. The main modification of the lumen environment caused by ICR is an increase in unabsorbed bile acids that induces an increase in the expression of genes responsible for bile salt uptake in colonocytes in the mouse ICR model [55]. We could hypothesize that the increase in bile acids in the intestinal lumen due to the loss of ileal reabsorption perturbs the lumen environment and contributes to the reduced diversity of small bowel and colonic microbiota, opening up niches that can then be occupied by bile resistant bacteria. In fact, 14 days after resection, the predominant phylotype identified in jejunum and colon was *Clostridium*, a genus containing many species capable of 7α-dehydroxylation of bile acids. In an environment where bile acids are increased, this bacterial group could potentially out-compete other members of the intestinal microbiota leading to higher concentrations of secondary bile acids. Interesting future directions include more direct analyses of bile acid, short chain fatty acid or lactate concentrations or pH of luminal contents to begin to define driving forces that shape residual microbiota after ICR

The present study uncovered a microbial dysbiosis caused by ICR and concomitant antibiotics. Common side effects of bowel resection including diarrhea, SIBO, and short bowel syndrome [56,57] may be linked to altered microbial diversity and composition. Antibiotics are already being tested as prophylaxis for post-surgical CD [58]. However, antibiotic therapy may damage beneficial as well as potentially detrimental commensal microbiota. Information about the biological characteristics of bacteria that can withstand the altered luminal environment after ICR is essential to rational design of alternative therapies, including probiotic or prebiotic therapies to improve outcome in post-surgical CD or SBS. The current study defines ICR-induced changes in microbiota in a murine model, and we have previously documented ability to perform ICR under germ-free conditions [27]. This will permit future studies aimed at colonization with specific microbiota after ICR to define those microorganisms that may survive in the altered luminal environment and potentially impact restoration of normal function.

In the present study we chose conventionally raised C57BL6 wild type mice after ICR as the initial step to elucidate the impact of ICR on the microbiota in remnant jejunum and proximal colon without the influence of the genetic background of the host or ongoing disease. However, a previous study by Rigby et al [27] showed that ICR results in persistent postsurgical inflammation in the small intestine and anastomosis of conventionalized IL10 null mice but not conventional wild-type animals at 28 days after ICR. Future experiments will include characterization of the post-ICR microbiota of hosts with genetic susceptibility to IBD, given that current results suggest that ICR dramatically alters the intestinal microbiota, which may contribute to risk of postsurgical inflammation.
